# Effects of alternating heat and cold stimulation at different cooling rates using a wearable thermo device on shoulder muscle stiffness: a cross-over study

**DOI:** 10.1186/s12891-022-05623-z

**Published:** 2022-07-14

**Authors:** Tomonori Sawada, Hiroki Okawara, Daisuke Nakashima, Shuhei Iwabuchi, Morio Matsumoto, Masaya Nakamura, Takeo Nagura

**Affiliations:** 1 Diagnosis and Treatment Division, Nagura Orthopedic Clinic, Chuo, Tokyo, Japan; 2 Department of Orthopaedic Surgery, Keio University School of Medicine, Shinjuku, Tokyo, Japan; 3 Department of Clinical Biomechanics, Keio University School of Medicine, Shinjuku, Tokyo, Japan

**Keywords:** Alternating heat and cold stimulation, Muscle hardness, Trapezius muscle, Skin temperature

## Abstract

**Background:**

A small, wearable thermo device that uses Peltier elements for programmed heat and cold stimulation has been developed recently and is expected to be applied in conventional contrast bath therapy. This study was aimed to examine improvements in trapezius muscle hardness and subjective symptoms resulting from alternating heat and cold stimulation, with different rates of cooling.

**Methods:**

This cross-over study included four conditions. Twenty healthy young male individuals (age, 22.3 ± 4.5 years) participated in this study. These four interventions targeted the unilateral trapezius muscle of the dominant arm after a 15-min typing task. Specifically, heat and cold stimulations were applied at different ratios (the heating/cooling rate of 3:1, 3:2, and 3:3) or not applied. Each intervention was separated by at least one week. Skin temperature at the stimulation area was recorded using a data logger. Outcome measures included muscle hardness (measured using a portable tester) and subjective symptoms (muscle stiffness and fatigue). Each item was assessed at three time points: baseline, after typing, and after the intervention.

**Results:**

Two-way analysis of variance with repeated measures found an interaction effect for muscle hardness between four conditions (3:1, 3:2, 3:3, and no) and three time points (*p* < 0.05). Only in the 3:1 condition were the post-intervention values lower than those after typing (*p* < 0.01). There was also an interaction effect for subjective muscle stiffness (*p* < 0.05); the values after the intervention in the 3:1 condition were lower than those after intervention in the no stimulation condition (*p* < 0.01). There was no significant relationship between changes in muscle hardness and changes in subjective symptoms in the 3:1 condition.

**Conclusions:**

Our results demonstrate that alternating heat and cold stimulations with a different cooling rate could affect the degree of improvement in muscle hardness and subjective symptoms. In particular, the 3:1 condition has the possibility to improved muscle hardness within the condition and subjective muscle stiffness between conditions.

**Trial registration:**

UMIN000040620. Registered 1 June 2020, https://upload.umin.ac.jp/cgi-open-bin/ctr_e/ctr_view.cgi?recptno=R000046359

**Supplementary Information:**

The online version contains supplementary material available at 10.1186/s12891-022-05623-z.

## Background

Since prehistoric times, humankind has learned from experience that hot and cold baths effectively alleviate pain and other symptoms; bathing has been commonly and widely used in health preservation and rehabilitation [1, 2]. The contrast bath is a method whereby alternating hot and cold water are applied. This is thought to cause intermittent vasoconstriction and vasodilation that induce a vascular pumping effect, promoting increased blood flow into the tissues. This provides oxygenation that improves healing, enhances waste product transportation (which reduces edema), improves limb function, and promotes a quicker recovery [3]. The contrast bath is used widely for recovery from fatigue after exercise, especially by athletes [4, 5]. However, it is limited by its requirement for a large bath, difficulty with water temperature control (given that it changes with each immersion), and hygiene problems (when multiple people use the same bath). Moreover, clear evidence has yet to be established, given that temperature settings, numbers of treatments, and durations of hot and cold water application have varied from one study to another [6–9].

Miniature apparatus using Peltier elements to programmatically control heat and cold stimulation can provide specific, rapid, and localized heating and cooling stimulation at increments of 0.1 °C and can potentially be widely used as alternatives to conventional contrast bath therapy. This enables optimal temperature protocol management to achieve its effect on the targeted muscle while preventing complications such as hot/cold burns. Such devices also have a further advantage in that the effects of temperature changes can be more accurately verified. In general, the muscle has been reported to become harder in pathological conditions, such as muscular damage, spasms, cramps, and edema [10–12]. Therefore, muscle hardness evaluation is considered useful to assess muscle fatigue associated with sustained muscle contraction. Recently, alternating heat and cold stimulations with this device has been to improve improved hardness in fatigued shoulder muscles better than heat stimulation alone [13]; the improvement in muscle hardness was associated with the degree of skin temperature cooling during stimulation. This suggested that, although 3:1 and 4:1 ratios are commonly used in conventional contrast bath therapy [3, 8], a prolonged cooling rate might be better with the wearable thermo device for improving hardness in fatigued muscles.

This study aimed to examine the effects of alternating heat and cold stimulation, with different rates of cooling, on the improvement of trapezius muscle hardness and subjective symptoms. Based on the results of our previous study, we hypothesized that, when using the thermo device, an increase in the cooling component (compared with the conventional contrast bath protocol) would be more effective in terms of the muscle hardness and subjective symptoms. Our results could contribute to establishing effective self-management protocols for using thermo devices for shoulder muscle stiffness or fatigue, which could assist many desk workers to maintain their health.

## Methods

### Study design

This was a single blinded, cross-over study registered with the UMIN Clinical Trials Registry (Registration number: UMIN000040620, date of first registration: 1/6/2020). The study protocol was conducted in compliance with ethical guidelines for medical and health research involving human subjects and was approved by the P-One Clinic Ethical Committee.

### Participants

Sample size calculation was performed with the G*Power 3.1.9.7 software, with 0.40 effect size (f), α = 0.05, and power (1-β) of 0.8, indicating a minimum of 20 participants. Participants were recruited from community and relevant universities through flyers and word of mouth in March 2021 by one clinic and its staff. Although inclusion criteria were volunteers without any orthopedic abnormalities of the neck and shoulders including males and females who agreed to participate in the study, as a result of this recruitment, a total of 20 healthy young males participated (Table 1). Exclusion criteria were individuals who refused to participate in the study who provided informed consent. All were informed of the study’s purpose and provided their informed consent prior to participation. The participants’ average daily smartphone use and average typing time over the past month were recorded by questionnaire.

**Table 1:** Participant characteristics (n = 20)

Age (years)	22.2 ± 4.4
Height (m)	1.76 ± 6.5
Weight (kg)	71.4 ± 11.8
BMI (kg/ m^2^)	22.9 ± 2.9
Dominant hand (n)	Right: 18, Left: 2
Time using smartphone per day (h)	4.8 ± 2.4
Typing time per day (h)	1.7 ± 1.6

Values are presented as mean ± standard deviation

### Experimental protocol

To compare the four intervention conditions, all participants were asked to visit our laboratory four times throughout the study. The room temperature within the laboratory was 24–26 °C. The Intervention in four conditions consisted of three different alternating heat and cold stimulation conditions and a no stimulation (NO) condition. The alternating heat and cold stimulation conditions were at ratios of 3 min of heat stimulation to 1 min of cold stimulation (H3C1), 3 min of heat stimulation to 2 min of cold stimulation (H3C2), and 3 min of heat stimulation to 3 min of cold stimulation (H3C3). To attenuate the order effects of intervention, participants were randomly and equally assigned to perform four interventions in the following four orders: 1) H3C1, H3C2, H3C3, and NO; 2) H3C2, H3C3, NO, and H3C1; 3) H3C3, NO, H3C1, and H3C2; and 4) NO, H3C1, H3C2, and H3C3. Each intervention was administered at least one week apart. The flow of each intervention day was kept identical, with each intervention being conducted after the typing task. The interventions and evaluations were performed on the unilateral upper trapezius muscle of the dominant hand—this muscle has been reported to be the most commonly affected by myofascial trigger points [14, 15]. First, trapezius muscle hardness and subjective symptoms were assessed. The participants then performed a 15-min typing task on a laptop to induce fatigue around the shoulder, following the method of a previous study [16]. The participants were instructed to keep the same posture while transcribing as much text as possible into document entry software. The texts used for the typing task were four out-of-copyright Japanese novels, displayed randomly to avoid duplication. After the typing task and before the intervention, muscle hardness and subjective symptoms were assessed. After the intervention, muscle hardness and subjective symptoms were assessed again.

### Intervention

We performed heat/cold stimulation using a commercially available, wearable thermo device (WTD) (REON POCKET 2; Sony Corporation, Tokyo, Japan). The WTD contains a Peltier element that uses voltage regulation to produce surface heating or cooling of an area of 4.5 × 5.5 cm. It can be operated using a smartphone application to provide repeated cooling, heating, and pausing for fixed numbers of seconds and the intensity can be adjusted in four levels from 1 to 4 for each of heating and cooling (level 1 as the weakest and level 4 as the strongest). The WTD was taped to the skin of the upper trapezius muscle. Heat stimulation was applied for 3 min, then cold stimulation for 1, 2, or 3 min, for a total five sets. Additionally, 10 s of movement cessation was allowed between heat and cold stimulations, to reduce thermal stress to the WTD. Performing five sets of alternating heat and cold stimulations in the three conditions required 22 min for H3C1, 27 min for H3C2, and 32 min for H3C3. The intensity of heat and cold stimulations was the same in all three stimulation conditions, with the heat stimulation set at level 3 and the cold stimulation at level 4. In the NO condition, the inactive WTD was applied for 20 min. During stimulation, participants rested in a relaxed position in a chair with a backrest.

### Measurements

Trapezius muscle hardness and skin temperature above it were assessed. The measurement point was 2 cm lateral to the midpoint between the 7^th^ cervical spinous process and the tip of the acromion [17, 18]. Muscle hardness was quantified using a portable muscle hardness meter (NEUTONE TDM-Z2; TRY-ALL, Chiba, Japan) by a trained examiner who was blind to the intervention conditions. A similar measurement device has been used in previous studies [19, 20]. In our previous study, we found the muscle hardness meter to have have excellent intra-tester reliability for the trapezius muscle (ICC_1,5_ = 0.992–0.995) [21]. The portable muscle hardness meter displays values on a scale of 0–100, without units. We converted the scale values to Newtons using the following formula, based on the manufacturer’s recommendation: *N* = 0.023 × measured value + 0.532. Measurements were obtained five times at each time point and the mean value was used for analysis.

For measuring subjective symptoms, participants were asked to rate the severity of muscle stiffness and fatigue using an 11-point numerical rating scale, with 0 indicating no stiffness/fatigue and 10 indicating the worst possible stiffness/fatigue. This method has also been utilized to assess stiffness and fatigue other than pain [22–24].

Muscle hardness and subjective symptomes were assessed at baseline, after the typing task, and after the intervention. During each intervention, the skin temperature of the stimulation area was measured using a thermocouple (JBS-7115-5 M-T; GRAPHTEC, Yokohama, Japan) and continuously recorded (at 1 Hz) by a data logger (midi LOGGER GL840; GRAPHTEC).

### Statistical analysis

Data are expressed as mean ± standard deviation. For comparing skin temperature changes in each intervention, we calculated the maximum and minimum temperature changes from the start of the intervention. A one-factor repeated measures analysis of variance (ANOVA) was performed to compare skin temperature changes between four conditions. When differences between conditions were detected, a Bonferroni correction was used for post hoc pairwise comparison, and p-values were multiplied by 6 because six different comparisons were performed. For trapezius muscle hardness and subjective symptoms data, two-way ANOVA with repeated measures was used to test the main effects at three time points (baseline, after typing, and after intervention) and four conditions (H3C1, H3C2, H3C3, and NO), and also the interaction effect between time point and condition. Bonferroni correction was performed for post hoc pairwise comparison, with p-values multiplied by 3 when comparing three measurement points within each condition, and by 6 when comparing four conditions at each measurement point. Where it was determined that an intervention promoted improvements in muscle hardness and subjective symptoms, correlations between and changes (after intervention minus after typing) in muscle hardness and subjective symptoms were assessed using Spearman’s correlation coefficient. All analyses were performed using SPSS Statistics version 27.0 (IBM, Armonk, NY, USA). Statistical significance was set at 0.05.

## Results

A total of 20 individuals participated in this study and were assessed for eligibility. Since no individual was excluded and dropped out during the study, the analysis was performed in all of them.

### Changes in skin temperature during the intervention

**Fig. 1:**
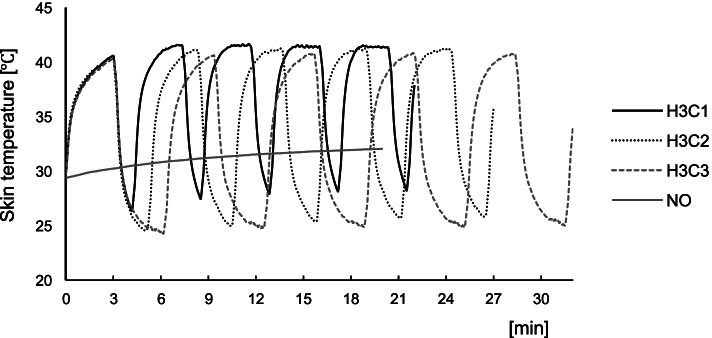
Mean skin temperature on the trapezius muscle in each condition. H3C1, alternating heat and cold stimulation at a ratio of 3:1. H3C2, alternating heat and cold stimulation at a ratio of 3:2. H3C3, alternating heat and cold stimulation at a ratio of 3:1. NO, no stimulation

**Fig. 2:**
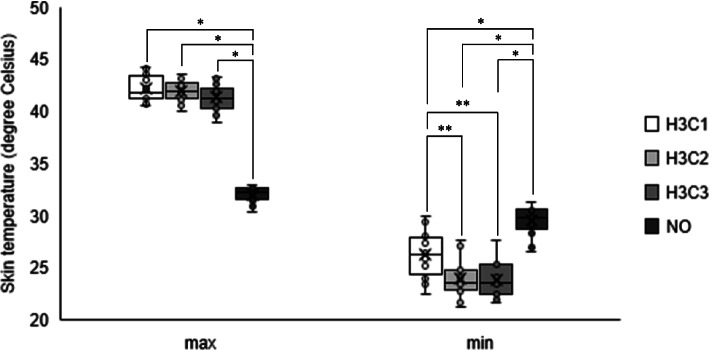
Average maximum and minimum skin temperatures in each condition. H3C1, alternating stimulation with 3 min of heat and 1 min of cooling. H3C2, alternating stimulation with 3 min of heat and 2 min of cooling. H3C3, alternating stimulation with 3 min of heat and 3 min of cooling. NO, no stimulation. * *p* < 0.01. ** *p* < 0.05

Figure 1 shows the mean skin temperatures during the intervention in each condition (the mean ± SD of skin temperature during each intervention is also shown in the Supplementary Material). Figure 2 shows the average maximum and minimum skin temperatures. The WTD’s alternating heat and cold stimulation was stable throughout the five sets of the intervention. As for the maximum skin temperature, a one-factor repeated measures ANOVA showed that the effect of condition was significant (F (3,57) = 389.500, *p* < 0.01). Post hoc analyses indicated significant differences between the three alternating conditions and the NO condition (*p* < 0.01, respectively) and the maximum temperature was approximately 10 °C higher in the three alternating conditions (H3C1, 42.2 ± 1.2 °C; H3C2, 41.9 ± 0.9 °C; and H3C3, 41.3 ± 1.1 °C) than in the NO condition (32.1 ± 0.7 °C). There was no statistically significant difference between the three intervention conditions. Similarly, as for the minimum skin temperature, the effect of condition was significant (F (3, 57) = 42.700, *p* < 0.01). Post hoc analyses indicated significant differences between the three alternating conditions and the NO condition (*p* < 0.01, respectively) and the minimum temperature was significantly lower in the three intervention conditions (H3C1, 26.3 ± 2.1 °C; H3C2, 24.0 ± 1.8 °C; and H3C3, 23.9 ± 1.6 °C) than in the NO condition (29.5 ± 1.3 °C). Furthermore, the H3C2 and H3C3 values were significantly lower than the H3C1 value (*p* < 0.05, respectively).

### Changes in muscle hardness

**Fig. 3:**
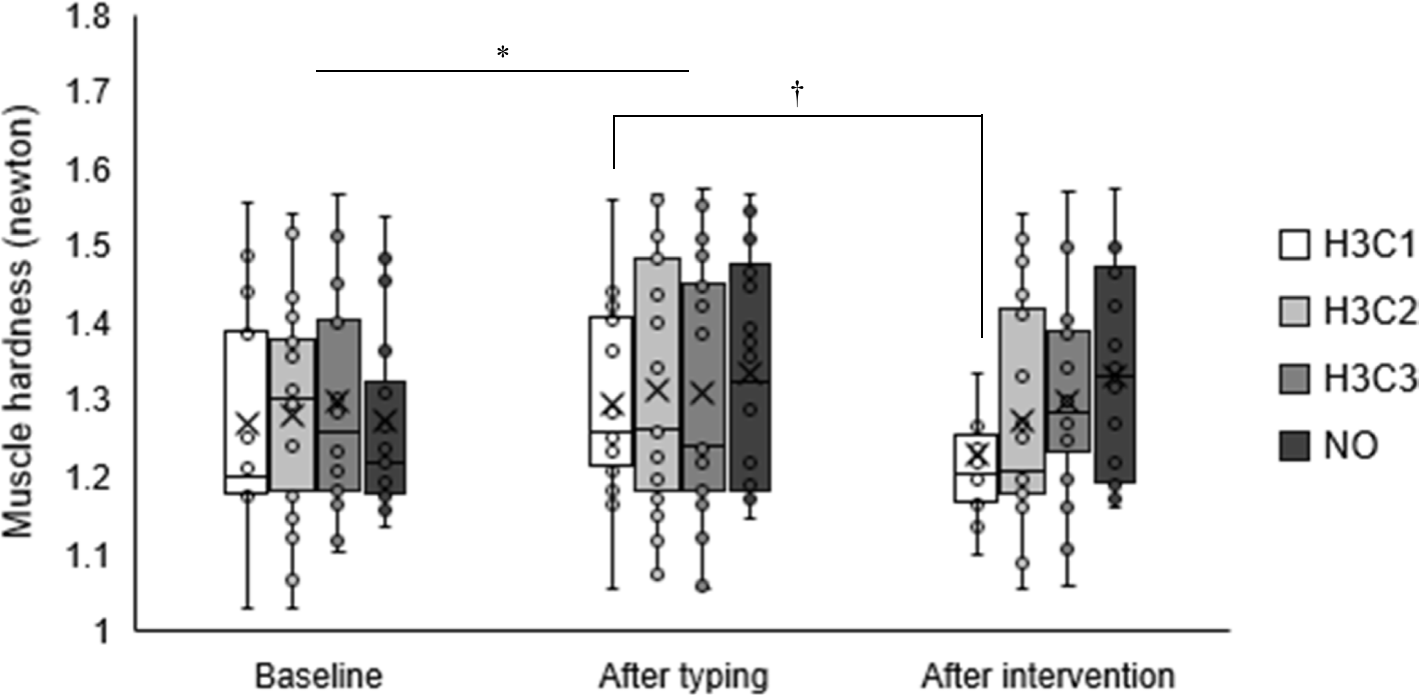
Mean trapezius muscle hardness values at baseline, after typing, and after intervention in each condition. H3C1, alternating stimulation with 3 min of heat and 1 min of cooling. H3C2, alternating stimulation with 3 min of heat and 2 min of cooling. H3C3, alternating stimulation with 3 min of heat and 3 min of cooling. NO, no stimulation. * There was a main effect of time point (*p* < 0.05) and a significant difference between baseline and after typing in multiple comparisons (*p* < 0.05). †: There was a significant interaction effect between time point and condition (*p* < 0.05) and a significant difference between after typing and after intervention in the H3C1 condition (*p* < 0.01)

Figure 3 and Table 2 show the mean trapezius muscle hardness values at baseline, after typing, and after intervention in the four conditions. There was a main effect of time point (F (2, 38) = 4.812, *p* < 0.05) and an interaction effect between time point and condition (F (2, 114) = 2.271, *p* < 0.05). A post hoc test showed that muscle hardness increased significantly after typing, compared with baseline (*p* < 0.05). Conversely, no statistically significant differences were observed between conditions for any three time points. Only in the H3C1 condition, muscle hardness significantly decreased after the intervention, compared with after typing (1.23 ± 0.13 N vs. 1.29 ± 0.12 N, respectively; F (2, 38) = 8.711, *p* < 0.01).

**Table 2:** Trapezius muscle hardness values at baseline, after typing, and after intervention in four conditions

	Baseline	After typing	After intervention
Trapezius muscle hardness [N]			
H3C1	1.27 ± 0.13	1.29 ± 0.12	1.23 ± 0.13
H3C2	1.28 ± 0.14	1.31 ± 0.16	1.27 ± 0.15
H3C3	1.30 ± 0.14	1.31 ± 0.16	1.30 ± 0.13
NO	1.27 ± 0.13	1.34 ± 0.15	1.33 ± 0.14

H3C1, alternating stimulation with 3 min of heat and 1 min of cooling. H3C2, alternating stimulation with 3 min of heat and 2 min of cooling. H3C3, alternating stimulation with 3 min of heat and 3 min of cooling. NO, no stimulation. Values are presented as mean ± standard deviation

### Changes in subjective symptoms

**Fig. 4:**
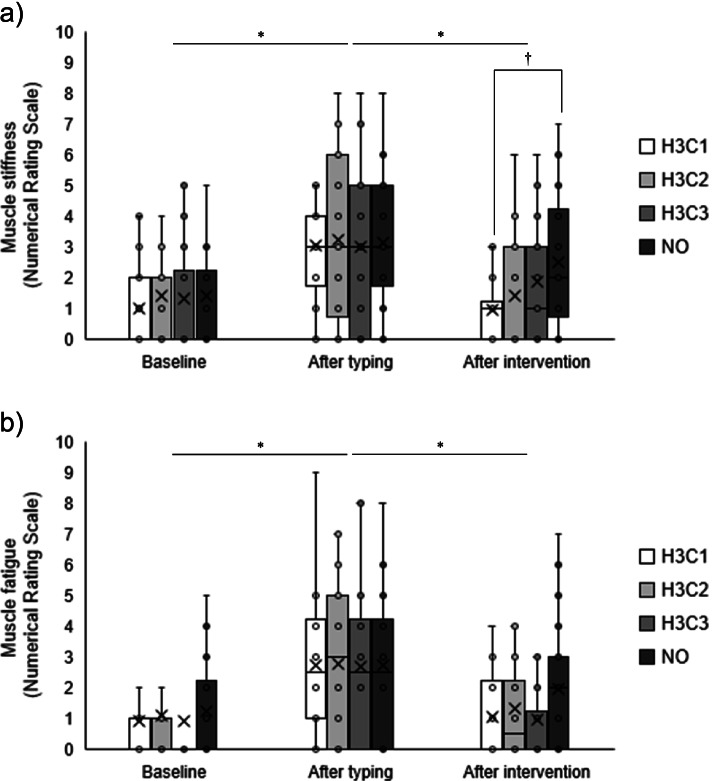
Mean subjective symptom values at baseline, after typing, and after intervention in each condition. **a** Muscle stiffness. **b** Muscle fatigue. H3C1, alternating stimulation with 3 min of heat and 1 min of cooling. H3C2, alternating stimulation with 3 min of heat and 2 min of cooling. H3C3, alternating stimulation with 3 min of heat and 3 min of cooling. NO, no stimulation. * There was a main effect of time point (*p* < 0.01) and a significant difference between baseline and after typing, and after typing and after intervention, in multiple comparisons (*p* < 0.01). †: There was a significant interaction effect between time point and condition (*p* < 0.01) and a significant difference between the H3C1 and NO conditions after the intervention (*p* < 0.01)

Figure 4 and Table 3 shows subjective symptom values at baseline, after typing, and after intervention in the four conditions. A main effect of time point was observed for muscle stiffness (F (2, 38) = 30.747, *p* < 0.01) and muscle fatigue (F (2, 38) = 21.198, *p* < 0.01). Values were significantly higher after typing, compared with baseline, and lower after the intervention, compared with after typing (*p* < 0.01). An interaction effect was also observed for muscle stiffness (F (6, 114) = 2.996, *p* < 0.01). The results of a post-hoc test for comparison between conditions for each time point and post-intervention values in the H3C1 condition were significantly lower than in the NO condition (1.0 ± 1.0 vs. 2.5 ± 2.1, respectively; F (3, 57) = 5.129, *p* < 0.01). Conversely, no statistically significant differences between conditions at the baseline and after typing muscle stiffness values. In other words, no fatigue accumulation in the previous experiment was observed at the baseline.

**Table 3:** Subjective symptom values at baseline, after typing, and after intervention in four conditions

	Baseline	After typing	After intervention
Muscle stiffness			
H3C1	1.0 ± 1.3	3.1 ± 1.9	1.0 ± 1.0
H3C2	1.4 ± 2.2	3.3 ± 2.6	1.5 ± 1.7
H3C3	1.4 ± 2.0	3.0 ± 2.5	1.9 ± 1.9
NO	1.4 ± 2.0	3.2 ± 2.1	2.5 ± 2.1
Muscle fatigue			
H3C1	0.9 ± 1.7	2.8 ± 2.2	1.1 ± 1.4
H3C2	1.2 ± 2.2	2.8 ± 2.6	1.4 ± 1.7
H3C3	0.9 ± 1.9	2.7 ± 2.7	1.0 ± 1.3
NO	1.3 ± 1.9	2.8 ± 2.3	2.0 ± 2.1

Values are presented as mean ± standard deviation

### Relationship between changes in muscle hardness and subjective symptoms

**Fig. 5:**
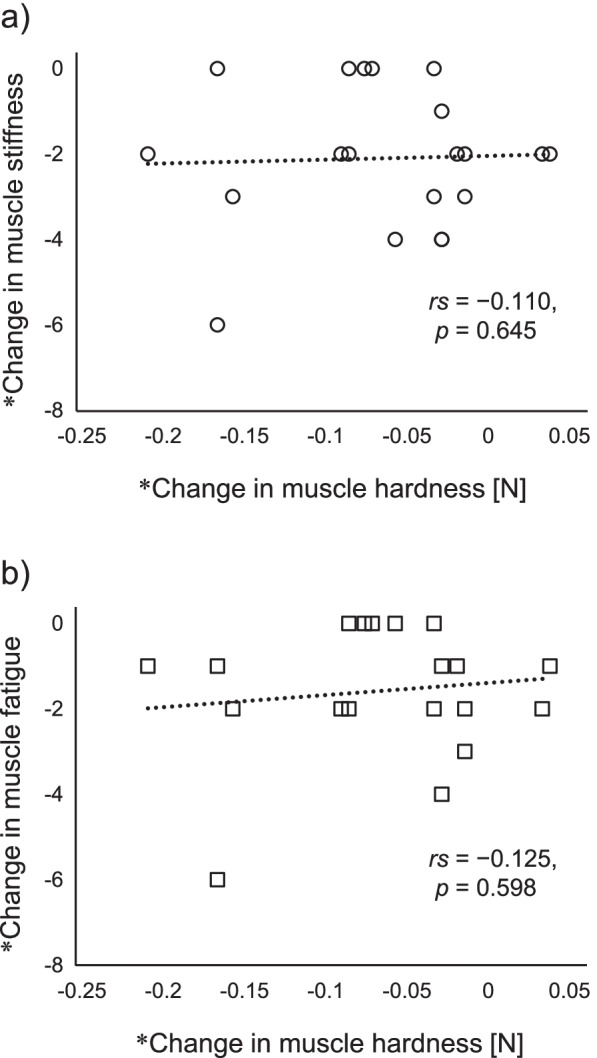
Scatter diagram representing changes in muscle hardness and subjective symptoms in the H3C1 condition. **a** Muscle stiffness. **b** Muscle fatigue. *The value was calculated as the after intervention value minus the after typing value

**Fig. 6:**
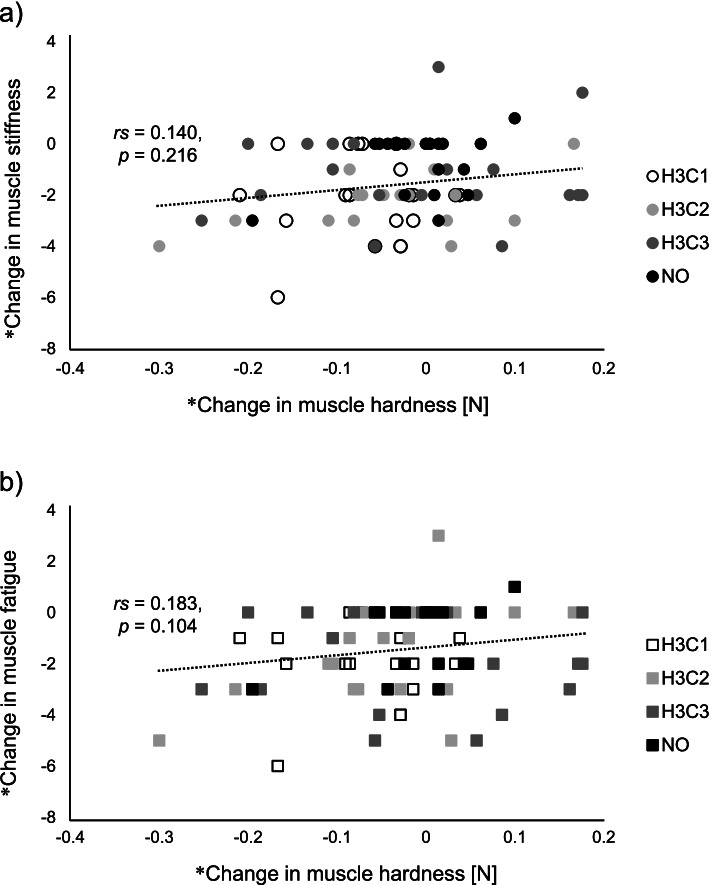
Scatter diagram representing changes in muscle hardness and subjective symptoms in each condition. **a** Muscle stiffness. **b** Muscle fatigue. H3C1, alternating stimulation with 3 min of heat and 1 min of cooling. H3C2, alternating stimulation with 3 min of heat and 2 min of cooling. H3C3, alternating stimulation with 3 min of heat and 3 min of cooling. NO, no stimulation. * The value was calculated as the after intervention value minus the after typing value

Because muscle hardness improved under the H3C1 condition, we investigated its association with subjective symptoms in the same condition (Fig. 5). However, we found no significant correlations between changes in muscle hardness and changes in subjective symptoms in the H3C1 condition, or in any of the other conditions (Fig. 6).

## Discussion

To the best of our knowledge, this is the first study to compare and validate the effects on shoulder muscle stiffness of multiple protocols of alternating heat and cold stimulation. The improvements in muscle hardness were obtained using the WTD in the H3C1 condition. Improvement in subjective symptoms (stiffness and fatigue) were obtained after the intervention, compared with after typing. Especially for muscle stiffness, a post hoc test showed that muscle stiffness improved significantly after intervention in the H3C1 condition, compared with the NO condition. However, in the H3C1 condition, the improvement in subjective symptoms did not correspond to changes in muscle hardness.

We examined the effects of alternating heat and cold stimulation on skin temperature of the local area using a data logger and confirmed that increases and decreases of skin temperature at the contact area occurred regularly according to the alternation protocol, which showed that the intervention could be implemented accurately. Temperature increase was similar in the three intervention conditions. However, temperature decrease was more pronounced in the 2-min and 3-min cooling protocols than in the 1-min cooling protocol, which suggests that cooling does not reach a plateau in the H3C1 condition.

Following a previous study [16], we set a typing task to induce muscle fatigue. Both trapezius muscle hardness and subjective symptoms increased after typing, compared with baseline. In our recent study [13], we performed a 30-min typing task to induce fatigue in the periarticular muscles of the shoulder joint and reported that there was no significant increase in trapezius muscle hardness before and after typing. We considered that a factor behind the lack of increase in muscle hardness was good posture during typing. Therefore, we selected a laptop computer that was likely to increase the user’s neck flexion angle [25–27]. Laptops are now more widespread than desktop computers. Therefore, our results may apply to desk workers who use laptop computers for long periods of time and are thus more prone to strain of cervical-to-shoulder muscles, including the trapezius muscle. An important finding of this study is that the general protocol used in conventional contrast bath therapy (3 min of heating and 1 min of cooling) was effective with the WTD for improving muscle hardness [13]. We reported in a recent study that alternating heat and cold stimulation was more effective than heat stimulation alone in improving muscle hardness. We also showed that improvements in muscle hardness were related to the degree and duration of skin temperature decrease from cold stimulation [13]. The results of the present study, in which the within-condition improvement was greater with 3:1 than with 3:2 or 3:3 heat/cold stimulation, suggest that the effects of thermal stimulation, such as improved blood flow in superficial tissues and extensibility of peripheral tissues including joints [28, 29], may be attenuated by a longer duration of decreased skin temperature. Fiscus et al. [30] examined blood flow in the lower leg during warm, cold, and contrast water therapy and reported that warm water therapy increased blood flow, compared with contrast water therapy. Therefore, to get the physiological effect derived from the thermal stimulation by local alternating heat and cold stimulation, it may be necessary to devise a protocol that increases the intensity of cooling in a short period of time, rather than a protocol that increases the cooling time as in the present study.

In addition to muscle hardness, we also examined the subjective parameters of muscle stiffness and fatigue. We found a main effect of time point for muscle stiffness and fatigue; they increased after typing and decreased after intervention. An interaction effect between time and condition was observed for muscle stiffness, with post-hoc tests showing muscle stiffness after the H3C1 intervention was significantly lower than those after NO condition. However, these subjective symptoms were not significantly associated with changes in muscle hardness. This indicates that improvements in muscle hardness are not necessarily reflected by improvements in subjective symptoms. Muscle hardness has been reported to increase in pathological conditions such as muscular damage, spasms, cramps, and edema [10–12]. Previous studies have shown that individuals with neck or shoulder pain have greater muscle hardness than asymptomatic participants [18, 31, 32]. However, there has been no association reported between muscle hardness and subjective muscle stiffness in individuals without pain [19, 21]. While a certain relationship between muscle hardness and pain exists, muscle stiffness and fatigue are more subjective complaints. The condition of localized muscle tissue may vary greatly among individuals.

This study has some limitations. First, we included only healthy adult male individuals (age, 19–38 years); the results cannot be applied to the general population, including women and patients with neck and shoulder pain. Second, because the participants were instructed that the four intervention conditions would be performed in four different orders, they could not be randomized and blinded with respect to following second and subsequent interventions. Third, the muscle hardness meter we used measured the value of muscle hardness not only from the target trapezius muscle but also the superficial skin, subcutaneous tissue, and deeper muscles. Fourth, there is a lack of clarity regarding the mechanisms underlying improved subjective muscle stiffness and fatigue. In future research, it will be necessary to include patients with neck and shoulder pain, evaluate blood flow in peripheral tissues, and examine the effects of heart rate variability on the autonomic nervous system.

## Conclusions

We demonstrated that alternating heat and cold stimulations with a different cooling rate could affect the degree of improvement in muscle hardness and subjective symptoms. In particular, the 3:1 condition improved muscle hardness within the condition and subjective muscle stiffness between conditions, suggesting that a conventional protocol may be more effective than a protocol with longer cooling times. However, the improvement in muscle hardness did not necessarily correspond to subjective improvements. In further research, it will be necessary to examine other heat/cold stimulation protocols with different intensities, and to establish an index for quantitative evaluation that reflects subjective improvements in muscle stiffness and fatigue.

## Supplementary Information

**Additional file 1: Supplemental Figure 1. **Mean skin temperature on the trapezius muscle during each condition (*n* = 20). (a) H3C1, alternating heat and cold stimulation at a ratio of 3:1. (b) H3C2, alternating heat and cold stimulation at a ratio of 3:2. (c) H3C3, alternating heat and cold stimulation at a ratio of 3:1. (d) NO, no stimulation. In each figure, the solid line shows the mean and the dashed line shows the mean ± standard deviation

## Data Availability

The datasets used and/or analysed during the current study are not publicly available due to disagreement of participants for their data to be shared but are available from the corresponding author on reasonable request.

## Abbreviations

ANOVA: Analysis of variance
WTD: Wearable thermo device
