# Supplementary material for: Effects of alternating heat and cold stimulation at different cooling rates using a wearable thermo device on shoulder muscle stiffness: a cross-over study
Source: BMC Musculoskelet Disord. 2022 Jul 14;23:669. doi: 10.1186/s12891-022-05623-z (PMC9281090; doi:10.1186/s12891-022-05623-z)
Supplement: Supplementary file 1 — Additional file 1: Supplemental Figure 1. Mean skin temperature on the trapezius muscle during each condition (n = 20). (a) H3C1, alternating heat and cold stimulation at a ratio of 3:1. (b) H3C2, alternating heat and cold stimulation at a ratio of 3:2. (c) H3C3, alternating heat and cold stimulation at a ratio of 3:1. (d) NO, no stimulation. In each figure, the solid line shows the mean and the dashed line shows the mean ± standard deviation [file 12891_2022_5623_MOESM1_ESM.pdf]

Supplementary Fig. 1

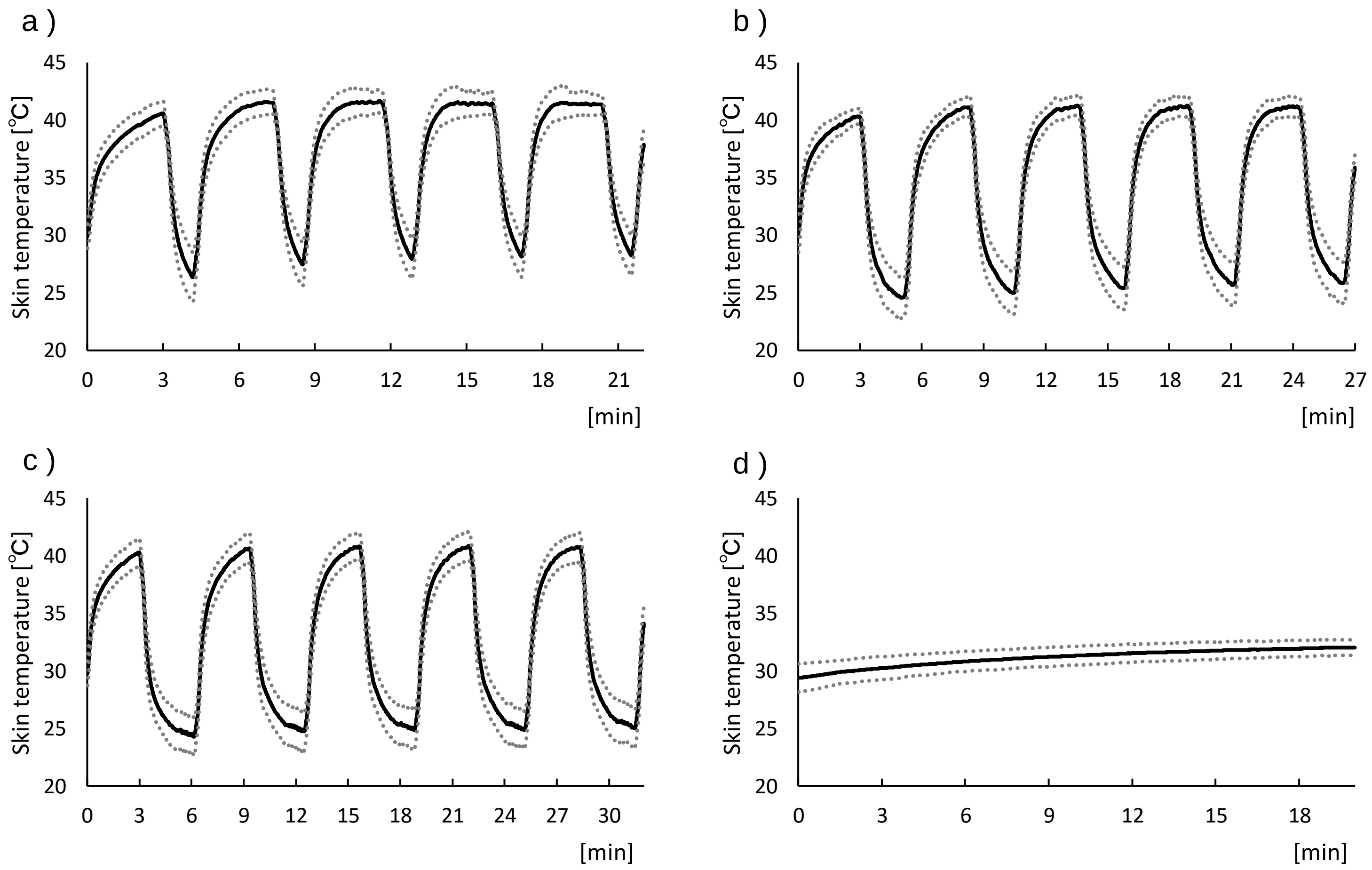

Supplemental Figure 1. Mean skin temperature on the trapezius muscle during each condition (n = 20)

(a) H3C1, alternating heat and cold stimulation at a ratio of 3:1. (b) H3C2, alternating heat and cold stimulation at a ratio of 3:2. (c) H3C3, alternating heat and cold stimulation at a ratio of 3:1. (d) NO, no stimulation. In each figure, the solid line shows the mean and the dashed line shows the mean ± standard deviation.
